# A Study on the Skin Whitening Activity of Digesta from Edible Bird’s Nest: A Mucin Glycoprotein

**DOI:** 10.3390/gels8010024

**Published:** 2021-12-28

**Authors:** Qunyan Fan, Jianmei Lian, Xuncai Liu, Fengyang Zou, Xin Wang, Maoshen Chen

**Affiliations:** 1 Xiamen Yanzhiwu Sinong Food Co., Ltd., Xiamen 360000, China; 15860770426@yanzhiwu.com (Q.F.); 13616007214@yanzhiwu.com (J.L.); 18988700208@yanzhiwu.com (X.L.); 13607550254@yanzhiwu.com (F.Z.); 2 State Key Laboratory of Food Science and Technology, Jiangnan University, Wuxi 214122, China; 6180111141@stu.jiangnan.edu.cn; 3 School of Food Science and Technology, Jiangnan University, Wuxi 214122, China; 4 International Joint Laboratory on Food Safety, Jiangnan University, Wuxi 214122, China

**Keywords:** edible bird’s nest, skin whitening activity, antioxidant activity, tyrosinase inhibitory activity

## Abstract

Edible bird’s nest (EBN) is an unusual mucin glycoprotein. In China, it is popular among consumers due to its skin whitening activity. However, the relationship between protein, sialic acid, and the whitening activity of EBN after digestion is still unclear. In the present work, the whitening activity (antioxidant activity and tyrosinase inhibitory activity) of digested EBN were studied by HepG2 and B16 cell models. The dissolution rate of protein and sialic acid was 49.59% and 46.45% after the simulated digestion, respectively. The contents of free sialic acid and glycan sialic acid in EBN digesta were 17.82% and 12.24%, respectively. HepG2 cell experiment showed that the digested EBN had significant antioxidant activity, with EC_50_ of 1.84 mg/mL, and had a protective effect on H_2_O_2_-induced oxidative damage cells. The results of H_2_O_2_-induced oxidative damage showed that the cell survival rate increased from 40% to 57.37% when the concentration of digested EBN was 1 mg/mL. The results of the B16 cell experiment showed that the digested EBN had a significant inhibitory effect on tyrosinase activity, and the EC_50_ value of tyrosinase activity was 7.22 mg/mL. Cell experiments showed that free sialic acid had stronger antioxidant activity and tyrosinase inhibitory activity than glycan sialic acid. The contribution rate analysis showed that protein component was the main antioxidant component in digestive products, and the contribution rate was 85.87%; free sialic acid was the main component that inhibited tyrosinase activity, accounting for 63.43%. The products of the complete digestion of EBN are suitable for the development of a new generation of whitening health products.

## 1. Introduction

Edible bird’s nest (EBN) is a traditional health food in China, which is a nest formed by mixing saliva and feather of Swiftlets [1]. The global EBN production is mainly concentrated in Southeast Asia, such as Indonesia, Thailand, Malaysia, with Indonesia’s EBN accounting for more than 80% of the world’s EBN production [2]. Two species of swiftlets (*Aerodramus* *fuciphagus* and *Aerodramus* *maximus*) build edible bird’s nests that are consumed by humans worldwide, as a delicacy known as the “Caviar of the East” [3]. The EBN was proven scientifically to possess high medicinal benefits in antioxidants [4], immune regulation, antihypertensive, anti-inflammatory, antiaging, and other biological activities [5,6]. EBN is well known for its remarkable skin whitening function [7,8].

The content of sialic acid in a bird’s nest is about 10% [9]. Sialic acid could inhibit tyrosinase activity, which was proposed to be a major component for the whitening function of EBN [10]. As tyrosinase, a rate-limiting enzyme in melanin biosynthesis catalyzes the hydroxylation of l-tyrosine to 3,4-dihydroxy-l-phenylalanine (l-dopa) (monophenolase reaction) and the subsequent oxidation of l-dopa to l-dopaquinone (diphenolase reaction). Thus, tyrosinase inhibitors have been proposed as skin-lightening agents [11]. Sialic acid can inhibit both mushroom and human tyrosinases in a dose-dependent manner. The IC_50_ of sialic acid on mushroom tyrosinase and human tyrosinase was 16.93 mM and 0.10 mM, respectively [12]. Sialic acid can reduce the melanin production of B16 mouse melanoma cells and A375 human melanoma cells. In the 3D human skin model (constructed by keratinocytes and melanocytes), sialic acid reduces the density of melanocytes in the skin, indicating that sialic acid can penetrate the keratinocyte layer and act on melanocytes, reducing the content of melanin [12]. Sialic acid usually combines with oligosaccharides, lipids, or proteins, while most of the sialic acid in EBN is in a conjugated form [13]. At present, studies have found that free sialic acid can inhibit tyrosinase activity and has a whitening function, but there are few reports about the activity of other forms of sialic acid.

Protein is another major part of EBN, which accounts for over 50% of the total dried weight of EBN [14]. The antioxidant peptides could be produced from edible bird’s nest protein hydrolysates [15]. Digested EBN showed stronger inhibition of melanogenesis of cultured B16 cells and enzymatic activity of tyrosinase, as compared with that of undigested EBN [16]. The whitening activity of skin is related to the formation of melanin. Excessive production of melanin can cause skin diseases such as freckles, old age spots, and chloasma [17]. Melanin takes l-tyrosine as a substrate, and under the catalysis of tyrosinase, melanin is generated through a series of reactions such as hydroxylation, oxidation, and intramolecular cyclization [18]. Therefore, the whitening activity of the samples can be evaluated by antioxidant activity and tyrosinase inhibitory activity [19].

The aim of this study is, therefore, to evaluate the skin whitening activity (antioxidant activity and intracellular tyrosinase inhibitory activity) of stewed EBN after simulated digestion by cell experiments (B16-F10 cells and HepG2 cells). The dissolution rate of protein, carbohydrate, and sialic acid, as well as the degree of protein hydrolysis during EBN digestion, were determined. The molecular weight (MW) distribution of peptide and the content of free and bound sialic acid in the digesta was measured. Meanwhile, the antioxidant activity and intracellular tyrosinase inhibitory activity of different forms of sialic acid were analyzed. The internal relationship between different components in digestive EBN and whitening activity was established, which provided a theoretical basis for further improving the whitening activity of EBN.

## 2. Materials and Methods

### 2.1. Materials

Pepsin, trypsin, sialic acid (SA), 2′,7′-dichlorofluorescin diacetate (DCFH-DA), 2,2′-azobis (2-amidinopropane) dihydrochloride (ABAP), and sialic acid (SA) were purchased from Sigma-Aldrich (St. Louis, MO, USA). Dulbecco’s modified Eagle’s medium (DMEM), fetal bovine serum (FBS), and penicillin–streptomycin were purchased from Shanghai Scientific & Technology Co., Ltd., Shanghai, China. Chromatography grade acetonitrile and tetrahydrofuran, analytical reagent of o-phenylenediamine hydrochloride (OPDH) and sulfuric acid were purchased from Sinopharm Chemical Reagent Co., Ltd, Shanghai, China. HepG2 and B16-F10 cells were kindly provided by Cell Bank, Chinese Academy of Sciences.

Dry EBN samples (*Aerodramus* *fuciphagus*) originated from Indonesia were kindly donated by Xiamen Yanzhiwu Sinong Food Co., Ltd. The contents of protein, carbohydrate, SA, and ash of EBN were 68.95%, 17.95%, 10.55%, and 2.15% (dry basis). EBN powder was prepared by a high-speed universal crusher (SS-1022, Shengshun, Jinhua, China) and then passed through a 60-mesh sieve. The EBN powder was stored in hermetically sealed glass bottles at room temperature before further analysis.

### 2.2. Digestion of EBN

Before digestion, stewed EBN solution was prepared. In brief, EBN powder (0.80 g) was steeped in 10 mL of deionized water in a 100 °C boiling water bath for 60 min. After cooling to room temperature, the stewed EBN solution was digested by a two-step in vitro digestion procedure, simulating gastric and small intestinal digestion. The digestion procedure has been reported by Minekus et al., with some reasonable modifications [20]. For this process, 10 mL of stewed EBN solution was added to a 50 mL centrifuge tube and then mixed with 7.5 mL of simulated gastric fluid (SGF) stock solution (containing 6.9 mM KCl, 0.9 mM KH_2_PO_4_, 25 mM NaHCO_3_, 47.2 mM NaCl, 0.1 mM MgCl_2_(H_2_O)_6_, 0.5 mM (NH_4_)_2_CO_3_ and 4000 U/mL pepsin). This was followed by the addition of 5 μL of 0.3 M CaCl_2_, before adjusting the pH to 2.0 with 1 M HCl. Finally, the volume of the gastric digestion was adjusted to 20 mL with distilled water. The mixture was incubated in a shaking water bath at 37 °C for 2 h.

Following the gastric phase simulation, the resulting chyme (20 mL) was mixed with 11 mL of simulated intestinal fluid (SIF) stock solution (containing 6.8 mM KCl, 0.8 mM KH_2_PO_4_, 85 mM NaHCO_3_, 38.4 mM NaCl, 0.33 mM MgCl_2_(H_2_O)_6_), 5.0 mL of pancreatin solution (800 U/mL) made up in SIF stock solution. Additionally, 40 μL of 0.3 M CaCl_2_ was added to the mixture. The pH of the intestinal digestion was adjusted to 7.0 and distilled water was added to achieve the desired final volume of 40 mL. The digestion process was then continued in the shaking water bath at 37 °C for 2 h [21].

During the in vitro digestion, nine centrifuge tubes were processed together. Each tube was taken at given time intervals of 0, 30, 60, 90, 120, 150, 180, 210, and 240 min, and enzymes were inactivated by incubating tubes in 100 °C boiling water for 10 min. The digesta were centrifugated at 1600× *g* for 10 min, and the supernatant was lyophilized for further analysis.

### 2.3. Dissolution Rate and Degree of Hydrolysis

The protein content of the digesta was determined by the Kjeldahl method, and the nitrogen (N) values were converted to protein content by N × 6.25. Carbohydrate determination was performed according to the phenol-sulfuric acid colorimetric method. The total content of SA in the digesta was determined described as below by high-performance liquid chromatography (HPLC) after hydrolysis and derivatization [22]. The dissolution rates of protein, carbohydrate, and SA were calculated. The degree of hydrolysis (DH) was determined according to the pH-stat method proposed in the study of Ding et al. [23]. All measurements were performed in triplicate.

### 2.4. Molecular Weight Distribution

The Mw distribution of the digesta was determined by an HPLC system (Agilent 1100, Palo Alto, CA, USA) using a TSK gel 2000 SWXL column (Tosoh, Shizuoka, Japan) [24]. The following conditions were used: sample volume of 10 μL, a mobile phase of acetonitrile/water/trifluoroacetic acid (45:55:0.1, *v/v/v*); a flow rate of 0.5 mL/min and ultraviolet detection wavelength of 220 nm.

### 2.5. Determination of Sialic Acid

After simulated digestion, sialic acids in free form, glycan form, and protein form were determined.

Freeform sialic acid (FFSA): In brief, digested hydrolysate (50 mg) was steeped in 40 mL deionized water with a magnetic stirring bar for 30 min at room temperature. Then, 1 mL solution was mixed with1 mL of 20 mg/mL OPDH solution for 40 min at 80 °C. The solution was cooled at room temperature and then centrifugated at 1600× *g* for 10 min. The supernatant was filtered through a 0.45 μm polytetrafluoroethylene filter (Agilent Technologies, Santa Clara, CA, USA). The content of FFSA was determined by HPLC.

Glycan-form sialic acid (GFSA): In brief, 1 mL digested hydrolysate solution was mixed with the same volume of Sevage reagent (Chloroform: N-butanol = 4:1, *v/v*) for 10 min at room temperature. After centrifugation at 1600× *g* for 10 min, the supernatant was extracted 3 times. The extracted aqueous phase layer was mixed with an equal volume of 2% (*v/v*) phosphoric acid solution and then hydrolyzed in a boiling water bath for 20 min. The supernatant was mixed with OPDH solution for derivatization. The content of SA was determined by HPLC. Finally, the content of GFSA was calculated as follows [22]:

GFSA% = the content of SA% − FFSA%

Protein-form sialic acid (PFSA): Firstly, the content of total SA was determined. In brief, digested hydrolysate (50 mg) was steeped in 40 mL of 2% (*v/v*) phosphoric acid solution and then hydrolyzed in a boiling water bath for 20 min. After centrifugation at 1600× *g* for 10 min, the supernatant was mixed with OPDH solution, and the content of total SA was determined. Finally, the content of PFSA was calculated as follows:

PFSA% = the content of total SA% − FFSA% − GFSA%

The content of SA after derivatization was determined by HPLC with an X-Bridge C18 column (4.6 mm × 250 mm, 5 µm). The mobile phase was 1.0% (*v/v*) tetrahydrofuran (containing 0.5% (*v/v)* phosphoric acid and 0.15% (*v/v*) N-butylamine)-acetonitrile (95:5, *v/v*). The excitation wavelength was 230 nm, and the emission wavelength was 425 nm. The column temperature was 35 °C, the flow rate was 1.0 mL/min, and the injection volume was 20 µL.

### 2.6. In Vitro Cytotoxicity Assay

The in vitro cytotoxicity assay was conducted by HepG2 cell and B16 cell. In brief, HepG2cells lines were seeded in 96-well plates to obtain an assay density of 5 × 10^4^ cells per well and cultured in DMEM medium containing 10% fetal bovine serum and 1% penicillin–streptomycin. B16 cells were seeded on 96-well plates with a density of 1 × 10^4^ per well [25]. The cells were cultured in 37 °C and a 5% CO_2_ incubator for 24 h to make them adhere to the wall completely. After 24 h, the cells were treated with different concentrations of digested EBN. After 24 h of exposure, the cytotoxic effects of digested EBN on cell lines were measured by using the MTT reduction experiment. The cell viability was calculated as follows:

$$\mathrm{Cell}\;\mathrm{viability}=\frac{\mathrm{Sample}\;\mathrm{Group}\;-\mathrm{Blank}\;\mathrm{Group}\;}{\mathrm{Control}\;\mathrm{Group}\;-\mathrm{Blank}\;\mathrm{Group}\;}\times 100\%$$

### 2.7. Cellular Antioxidant Activity

The cellular antioxidant assay (CAA) of digested EBN was determined according to the method described by Wolfe and Liu [26], with some modifications. In brief, HepG2 cells were incubated in DMEM containing 10% FBS and 1% penicillin–streptomycin at 37 °C in a 90% humidity and 5% CO_2_ incubator. HepG2 cells were seeded at a density of 5 × 10^4^ cells/well onto 96-well plates and incubated. After 24 h incubation, the media was removed and the cells were washed with PBS buffer. Triplicate wells were treated with 100 mL of digested EBN in treatment media for 24 h at 37 °C. The wells were washed with PBS and 100 mL of 25 µM DCFH-DA in treatment medium was added and incubated for 1 h. Then, the cells were washed with PBS and 100 mL 600 µM ABAP was applied to the cells. Immediately following, the 96-well plate was placed into a fluorescence microplate instrument. The fluorescence was recorded at 535 nm (emission) and 485 nm (excitation) every 5 min for 1 h at 37 °C. Control wells were treated with DCFH-DA and ABAP and blank wells were treated with DCFH-DA without ABAP. The area under the curve of fluorescence versus time was integrated to calculate the CAA value [27].

$$\mathrm{CAA}\;\mathrm{Value}=(1-\frac{\int \mathrm{SA}}{\int \mathrm{CA}})\times 100\%$$

where ∫SA and ∫CA are the integral areas of the fluorescence curve of the sample group and control group, respectively. EC_50_ is obtained from the median effect curve of the log$\frac{fa}{fu}$ and log (does), where *fa* is the curve area affected by the sample, *fu* is the curve area not affected by the sample, and log (does) is the logarithm of the sample concentration. When log$\frac{fa}{fu}\;$= log1 = 0, the EC_50_ is obtained.

### 2.8. Hydrogen-Peroxide-Induced Cell Oxidative Damage

The cells were prepared into 5 × 10^5^ cells/mL single-cell suspension, inoculated with 100 μL in each well of 96-well plates, cultured at 37 °C for 24 h, and the medium was removed. Different concentrations of sample solutions diluted with culture medium were added and cultured at 37 °C for 4 h. The medium was then removed, and 300 μM H_2_O_2_ was added and incubated for 2 h. The MTT method was used to read the absorbance at 570 nm and calculate the cell survival rate [28].

### 2.9. Intracellular Tyrosinase Activity Assay

Intracellular tyrosinase activity was evaluated by the rate of dopaquinone formation from tyrosine [29]. The logarithmic growth phase B16 cells were seeded on 96-well plates, with a density of about 1 × 10^4^ cells/well, and each well was inoculated with 100 μL. Cells were cultured in 37 °C and a 5% CO_2_ incubator for 24 h, to make them adhere to the wall completely. Remove the culture medium and wash it with PBS. Different concentrations of the sample solution were added, diluted by the culture medium, and incubated in a 37 °C incubator for 24 h. The culture medium was removed and washed with PBS. In each well, 100 μL of Triton X-100 solution was added, containing 1% volume fraction, the enzyme plate was sealed with sealing film and placed in the refrigerator at −80 °C for 60 min. Then, the culture plate was taken out and placed at room temperature. The culture plate was preheated at 37 °C for 1 h, and 10 μL of 1 mg/mL l-tyrosine solution was added. The solution was then measured at 475 nm.

$$\mathrm{Tyrosinase}\;\mathrm{inhibitory}\;\mathrm{activity}=\frac{\mathrm{Sample}\;\mathrm{Group}\;-\mathrm{Blank}\;\mathrm{Group}\;}{\mathrm{Control}\;\mathrm{Group}\;-\mathrm{Blank}\;\mathrm{Group}\;}\times 100\%$$

### 2.10. Contribution Rate of Component

The contribution rate (CR) of FFSA, GFSA, and protein components in the digesta was calculated as follows:

$$\mathrm{CR}\;\mathrm{of}\;\mathrm{FFSA}=\frac{{\mathrm{EC}}_{50}\;\mathrm{of}\;\mathrm{digesta}\times \mathrm{the}\;\mathrm{content}\;\mathrm{of}\;\mathrm{FFSA}}{{\mathrm{EC}}_{50}\;\mathrm{of}\;\mathrm{FFSA}}\times 100\%$$

$$\mathrm{CR}\;\mathrm{of}\;\mathrm{GFSA}=\frac{{\mathrm{EC}}_{50}\;\mathrm{of}\;\mathrm{digesta}\times \mathrm{the}\;\mathrm{content}\;\mathrm{of}\;\mathrm{GFSA}}{{\mathrm{EC}}_{50}\;\mathrm{of}\;\mathrm{GFSA}}\times 100\%$$

CR of Protein component %=100−CR of FFSA %−CR of GFSA %

### 2.11. Data Analysis

Data analysis was carried out using one-way analysis of variance (ANOVA) with Duncan’s multiple range test and the general linear model (GLM), using the multivariate test with Statistical Package for SPSS software version 12. The differences in means between the samples were determined at the 5% confidence level (*p* < 0.05).

## 3. Results and Discussion

### 3.1. Analysis of the Physicochemical Properties of Digested EBN

#### 3.1.1. Dissolution Rate and Degree of Hydrolysis

**Figure 1:**
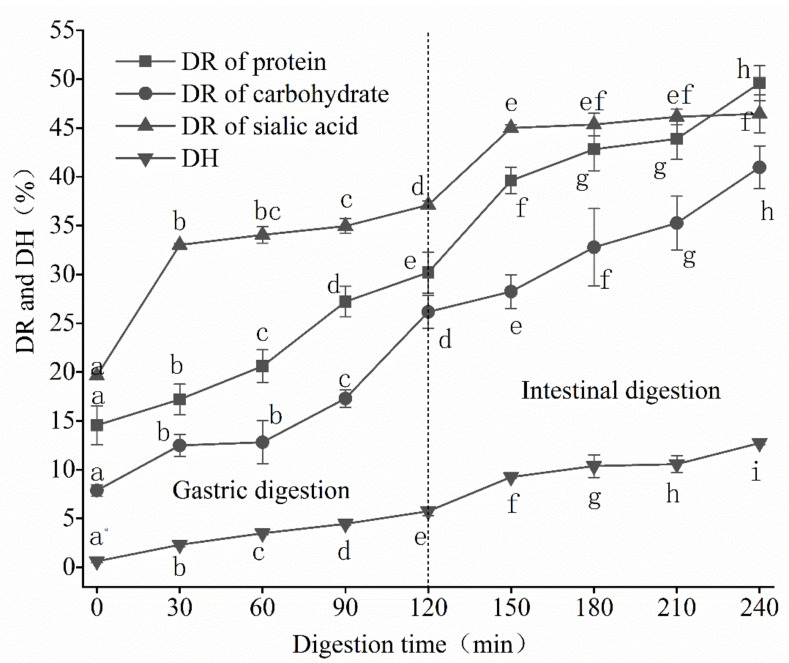
Dissolution rate (DR) of protein, carbohydrate, and sialic acid, as well as the degree of protein hydrolysis (DH) of digesta during digestion. The different superscript letters for each index represent significant differences (*p <* 0.05). (mean ± STD, *n* = 3).

The sialic acid and glycoprotein of EBN have a complex structure and are not easy to dissolve in water [30]. The dissolution rate of protein, carbohydrate, and sialic acid during EBN digestion is displayed in Figure 1. At the beginning of digestion, the protein dissolution rate was 14.54%, then it increased to 30.19% after the stomach stage, and further enhanced to 49.59% after the intestine stage. For carbohydrates, the dissolution rate increased from 7.86% initially to 26.17% after gastric digestion and 40.97% after intestinal digestion. During the whole digestion process, the dissolution rate of protein and carbohydrate exhibited a significant linear positive correlation (r = 0.98, *p* < 0.01). The sugar chains and protein in the EBN protein were dissolved into the digestion supernatant together. However, the dissolution rate of sialic acid is significantly different. Specifically, the dissolution rate of sialic acid increased rapidly, from the initial 19.62% to 33.025%, within 30 min of gastric digestion, and then slowly increased to 37.11% after 2 h of gastric digestion. In the intestinal stage, the dissolution rate of sialic acid showed a similar trend to that in the stomach stage. The dissolution rate of sialic acid rapidly increased to 44.99% within the first 30 min of the intestinal stage and then increased gently to 46.45% after 2 h. The degree of breakage in protein–peptide bonds is characterized by the degree of hydrolysis. It could be seen from Figure 1 that the degrees of EBN hydrolysis in the stomach and intestine stage were 5.75% and 12.72%, respectively, suggesting that the hydrolysis of protein mainly occurred in the intestinal stage, which is attributed to the that the trypsin is beneficial to promote the dissolution rate of EBN protein [31].

#### 3.1.2. Mw Distribution

**Figure 2:**
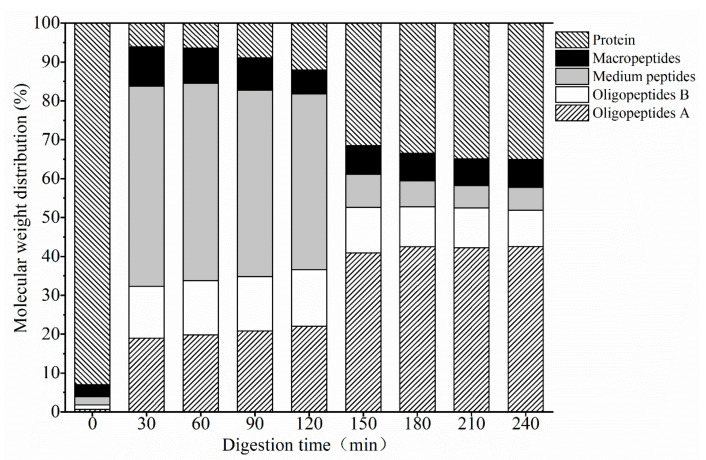
The molecular weight distribution of digesta during digestion: protein: >10 kDa; macropeptides: 5–10 kDa; medium peptides: 1–5 kDa; oligopeptides B: 0.5–1 kDa; oligopeptides A: < 0.5 kDa.

Peptides are easier to be absorbed and utilized by the human body than intact proteins. The digested products of the EBN protein are divided into oligopeptides (<1 kDa), medium peptides (1~5 kDa), macropeptides (5–10 kDa), and proteins (>10 kDa), based on their relative molecular weight. As indicated in Figure 2, at the beginning of digestion, it was mainly macromolecular sialoglycoprotein in the stewed EBN solution, and the relative content of protein exceeded 93%. Within 30 min of gastric digestion, the water-soluble protein in EBN was rapidly hydrolyzed into oligopeptides and medium molecular weight peptides, while the number of macropeptides and proteins was relatively low. Additionally, after 30 min, the relative distribution of peptides stabilized, the relative content of oligopeptides and proteins increased slightly, and the relative content of medium-MW and macro-MW peptides decreased. After gastric digestion for 2 h, the relative content of oligopeptides, medium peptides, and macropeptides were 36.62%, 45.16%, and 6.17%, respectively. Among them, the relative content of oligopeptides with an MW less than 500 Da was 22.02%, and the relative content of protein was 12.05%. The protein with an MW of 128 and 106 kDa was the highest protein content among water-soluble EBN proteins. The isoelectric point of both proteins was ≤3 [11]. It is speculated that the pH of the digestive juice changes from acidic to neutral after entering the pancreatic stage; thus, the protein is more easily dissolved, and gastric enzymes and pancreatin promote the dissolution of the protein. The relative distribution of peptides changed significantly within 30 min of intestinal digestion. The relative content of oligopeptides and protein increased by 16.02% and 19.40%, respectively, and the medium peptides decreased by 36.67%, while the relative content of macropeptides did not change significantly, and the relative distribution of peptides stabilized after 30 min. After 2 h of intestinal digestion, the relative contents of oligopeptides, medium peptides, and macropeptides were 52.88%, 5.86%, and 7.15%, respectively. The relative content of <500 Da oligopeptides was 42.55%, and the relative content of protein was 34.11%. Intestinal digestion may promote the hydrolysis of medium peptides into oligopeptides. The results implied that the polypeptides in the digested products of EBN were mainly oligopeptides.

#### 3.1.3. Determination of Sialic Acid

**Figure 3:**
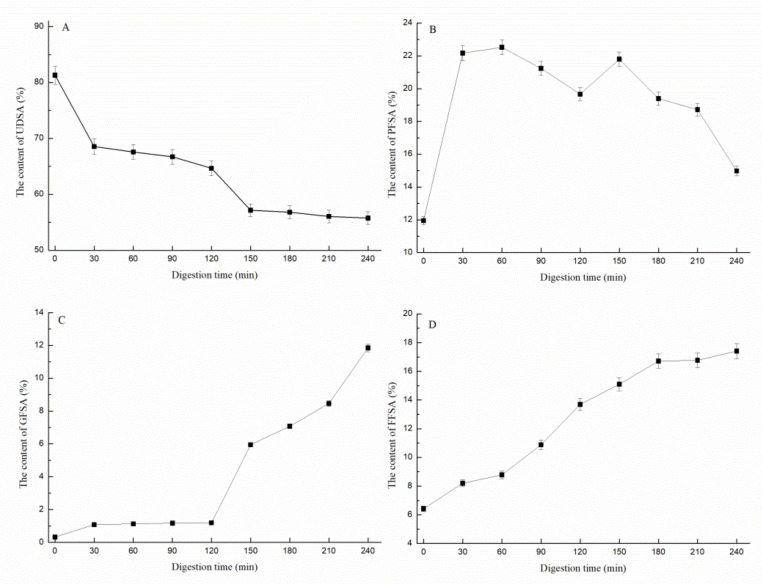
The relative percentage of UDSA (**A**), PFSA (**B**), GFSA (**C**), and FFSA (**D**) during digestion: UDSA: undigested sialic acid; PFSA: protein-form sialic acid; GFSA: glycan-form sialic acid; FFSA: freeform sialic acid.

The sialic acid in the EBN is mainly bound to the sugar chain end of glycoprotein. The clinical results of infants and young children showed that the oligosaccharides containing sialic acid in breast milk are more beneficial to the brain development of young children [32]. Sialic acid, combined with oligosaccharides or peptides, has a higher biological effect [33]. The relative percentage of different forms of sialic acid during EBN digestion is shown in Figure 3. At the beginning of digestion, the relative content of FFSA was 6.82%, which reached 14.09% after 2 h of gastric digestion. In the stomach stage, the relative content of GFSA increased gradually, and the relative content of GFSA was always maintained at less than 2%, while the relative content of PFSA increased by 10% within 30 min before the digestion and then stabilized at 22%. After 2 h of gastric digestion, 63.46% of undissolved sialic acid was still bound in the digested centrifugal protein. The pancreatic stage was conducive to the production of GFSA. After gastrointestinal digestion, 17.82% of the sialic acid was present in free form, 12.24% was bound to glycans, 15.39% was bound to proteins, and 54.55% was still insoluble in the digestion supernatant.

### 3.2. Whitening Activity of Digested EBN

#### 3.2.1. In Vitro Cytotoxicity and Protective Effect of Digested EBN on HepG2 Cell Viability

**Figure 4:**
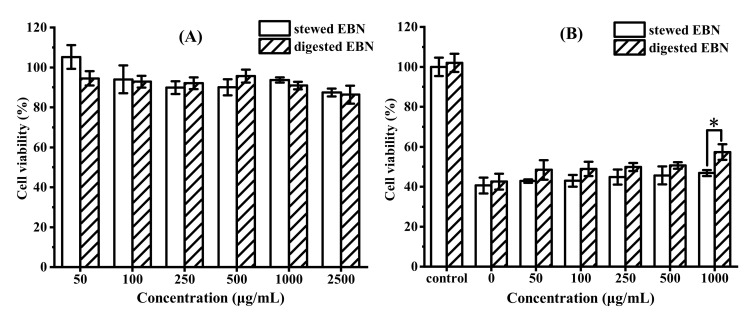
(**A**) HepG2 cytotoxicity of stewed and digested EBN at different concentrations and (**B**) the protective effects of stewed and digested EBN on HepG2 cells subjected to H_2_O_2_-induced oxidative damage. (mean ± STD, *n* = 3; * *p* < 0.05).

The safe concentration of digested EBN on HepG2 cells was determined by MTT assay, and the results are shown in Figure 4A. When the concentration of digested EBN was lower than 1 mg/mL, the cell viability was more than 90%, and it was non-toxic to HepG2 cells, which was a safe concentration for cell antioxidant experiments. Reactive oxygen species such as H_2_O_2_ may change the structure of biological macromolecules, leading to cytotoxicity and damage. Oxidative stress and the increase in reactive oxygen species are key factors of aging [34]. As H_2_O_2_ is the main precursor of highly reactive oxygen species, this study used it to induce oxidative damage of HepG2 cells. HepG2 cells were induced oxidative damage by H_2_O_2_ to study the protective effect of digested EBN on ROS-mediated cell damage [15]. The results are shown in Figure 4B. Compared with the control group, the cell viability treated with H_2_O_2_ for 2 h decreased to 40%. When the concentration of stewed EBN without digestion was 1 mg/mL, the cell viability was only 46.85%. The molecular weight of the EBN protein was too large to enter the cells directly to protect the cells. After digestion, the protective effect of stewed EBN on oxidative damage cells was significantly improved (*p* < 0.05). When the concentration of digested EBN was 1 mg/mL, the cell survival rate was increased to 57.37%.

#### 3.2.2. CAA of Digested EBN

**Figure 5:**
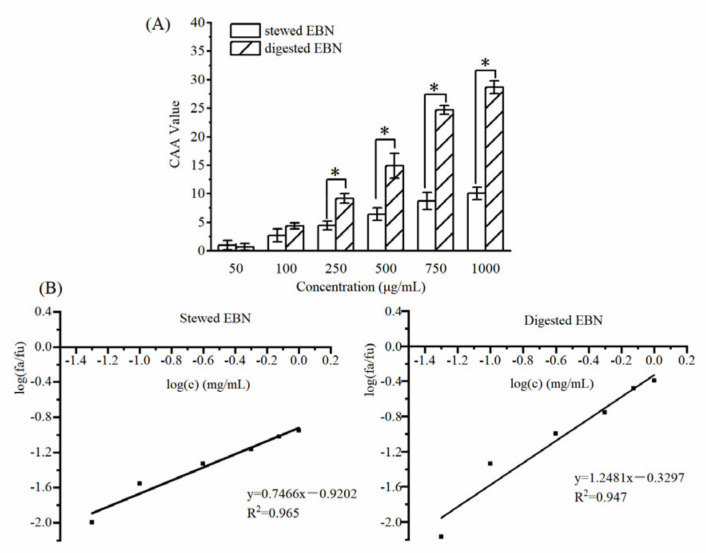
Cellular antioxidant activities of stewed and digested EBN at different concentrations (**A**). Determination of the EC_50_ of the stewed EBN and digested EBN (**B**). (mean ± STD, *n* = 3; * *p* < 0.05).

The results of the intracellular antioxidant activity experiment of digested EBN are shown in Figure 5A. When the concentration was higher than 0.25 mg/mL, the CAA value of digested EBN was significantly higher than that of stewed EBN (*p* < 0.05). Digestion promoted the absorption of EBN and increased the antioxidant activity of EBN. The scavenging ability of digested EBN on peroxy radicals in HepG2 cells was significantly improved. According to the linear regression curve in Figure 5B, the EC_50_ value of cell antioxidant activity can be calculated. The EC_50_ value of stewed EBN was 17.08 mg/mL, and the EC_50_ value of digested EBN was 1.84 mg/mL.

#### 3.2.3. In Vitro Cytotoxicity and Intracellular Tyrosinase Inhibition of Digested EBN on B16 Cell Viability

**Figure 6:**
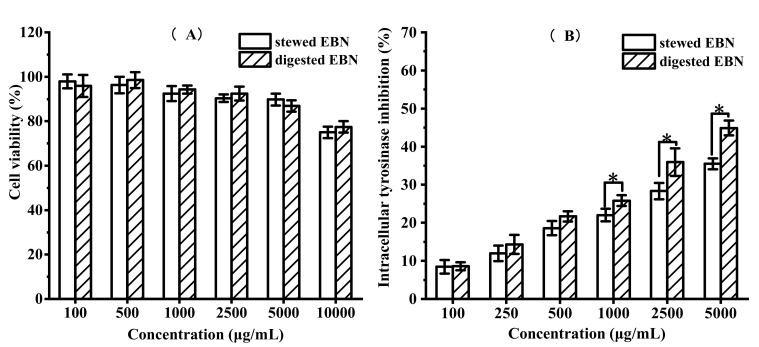
(**A**) B16 cytotoxicity of stewed and digested EBN at different concentrations and (**B**) the intracellular tyrosinase inhibition of stewed and digested EBN. (mean ± STD, *n* = 3; * *p* < 0.05).

To further study the whitening activity of EBN, the MTT method and intracellular tyrosinase activity experiment were used to evaluate the whitening activity of stewed EBN and digested EBN. MTT assay was used to determine the safe concentration of stewed EBN and digested EBN on B16 cells. The cytotoxicity and intracellular tyrosinase inhibition were detected and the results are shown in Figure 6. When the concentration of stewed EBN and digested EBN was less than 5 mg/mL, the cell viability was more than 90%, which was non-toxic to B16 cells. This concentration was a safe concentration for the inhibition of cell tyrosinase activity. The inhibitory effect of stewed EBN and digested EBN on tyrosinase activity in B16 cells is shown in Figure 6B. The intracellular tyrosinase inhibitory activity of digested EBN was significantly higher than that of stewed EBN. The EC_50_ value of tyrosinase activity of stewed EBN was 18.74 mg/mL, and the EC_50_ value of digested EBN was 7.22 mg/mL.

### 3.3. Mechanism Analysis of Whitening Activity

#### 3.3.1. In Vitro Cytotoxicity and Protective Effect of Sialic Acid on HepG2 Cell Viability

**Figure 7:**
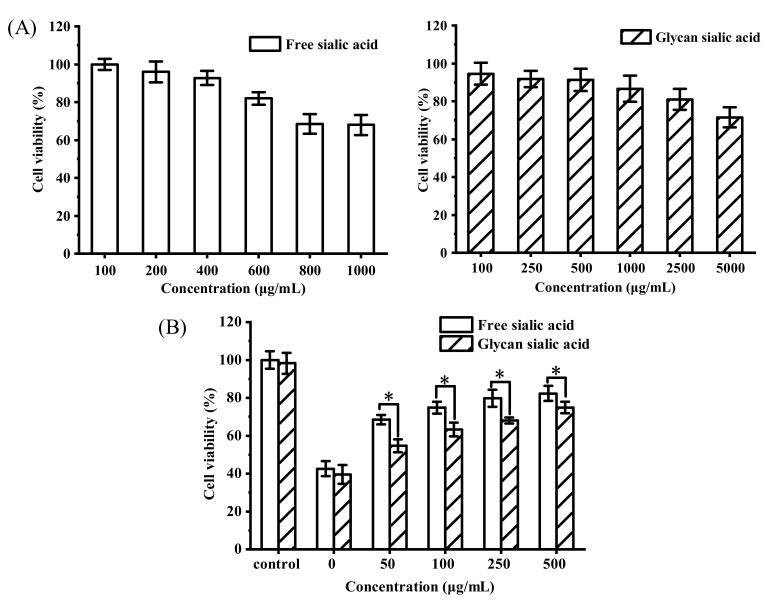
(**A**) HepG2 cytotoxicity of free sialic acid and glycan sialic acid at different concentrations, and (**B**) the protective effects of free sialic acid and glycan sialic acid on HepG2 cells subjected to H_2_O_2_-induced oxidative damage. (mean ± STD, *n* = 3; * *p* < 0.05).

The digested EBN contains sialic acid and protein components, in which sialic acid exists in free, glycan, and protein forms, while protein components contain amino acids, peptides, and partially hydrolyzed proteins. Sialic acid was an important active component in EBN. The safe concentrations of free sialic acid and glycan sialic acid on HepG2 cells were determined by MTT assay. The results are shown in Figure 7A. When the concentration of free sialic acid was less than 0.5 mg/mL, the cell viability was more than 90%, and it was non-toxic to HepG2 cells. This concentration was a safe concentration for the cell antioxidant experiment. When the concentration of glycan sialic acid was less than 1 mg/mL, the cell viability was more than 85%, and the cytotoxicity of glycan sialic acid was lower than that of free sialic acid. The protective effects of free sialic acid and glycan sialic acid on ROS oxidative damage cells were studied. The results are shown in Figure 7B. Compared with the control group, the cell viability treated with H_2_O_2_ decreased to 40% after 2 h. Both free sialic acid and glycan sialic acid had protective effects on HepG2 cells damaged by oxidation. The protective effect of free sialic acid on oxidative damage cells was significantly higher than that of glycan sialic acid (*p* < 0.05). After pretreatment with 0.5 mg/mL of free sialic acid and glycan sialic acid, HepG2 cell viability increased to 82% and 74%, respectively.

#### 3.3.2. CAA of Sialic Acid

**Figure 8:**
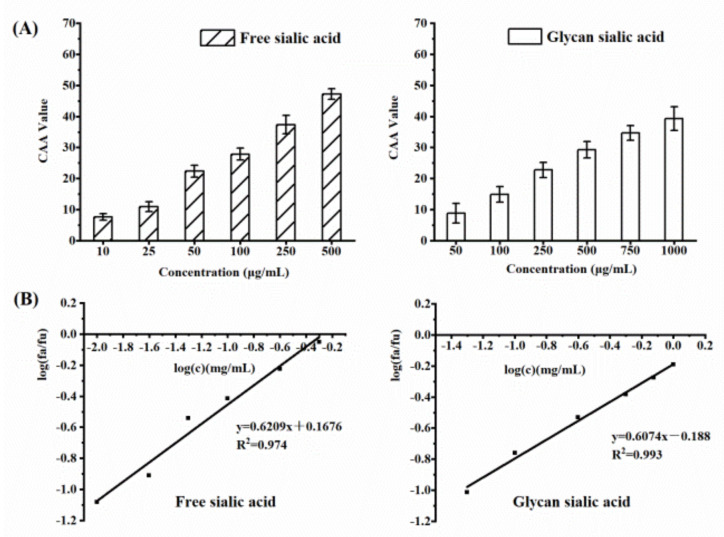
Cellular antioxidant activities of free sialic acid and glycan sialic acid at different concentrations (**A**). Determination of the EC_50_ of the free sialic acid and glycan sialic acid (**B**). (mean ± STD, *n* = 3).

The results of the cell antioxidant activity test are shown in Figure 8A. The CAA value of free and glycan sialic acids increased with the increase in concentration, and they can be absorbed by cells to exert antioxidant activity. The EC_50_ value of the antioxidant activity is shown in Figure 8B. The EC_50_ values of free sialic acid and glycan sialic acid were 0.54 mg/mL and 2.04 mg/mL, respectively. The antioxidant activity of free sialic acid was significantly higher than that of glycan sialic acid (*p* < 0.05).

#### 3.3.3. In Vitro Cytotoxicity and Intracellular Tyrosinase Inhibition of Sialic Acid on B16 Cell Viability

**Figure 9:**
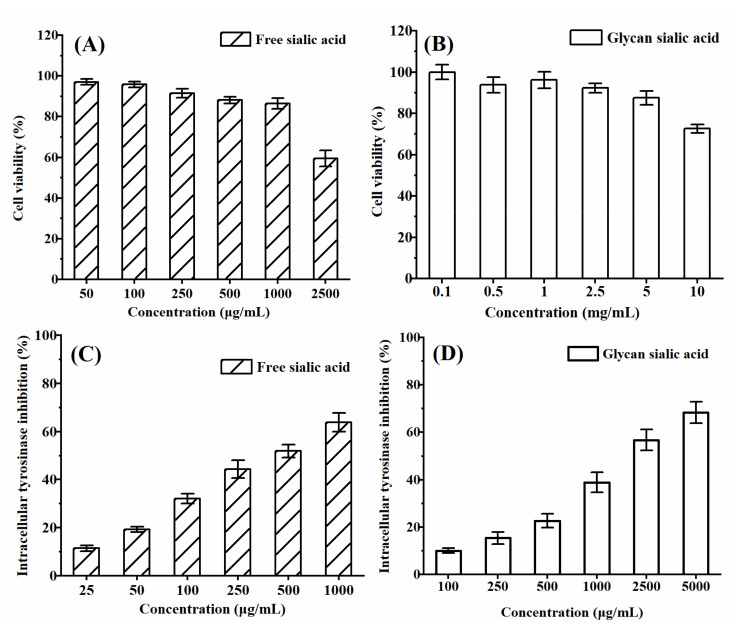
B16 cytotoxicity of free sialic acid (**A**) and glycan sialic acid (**B**) at different concentrations and the intracellular tyrosinase inhibition of free sialic acid (**C**) and glycan sialic acid (**D**). (mean ± STD, *n* = 3).

The safe concentrations of free sialic acid and glycan sialic acid on B16 cells were determined by MTT assay. The cell viability and intracellular tyrosinase inhibition were analyzed and the results are shown in Figure 9. When the concentration of free sialic acid was less than 1 mg/mL, the cell viability was more than 90%. When the concentration of glycan sialic acid was less than 5 mg/mL, the cell viability was more than 90%. The biological safety of glycan sialic acid was higher than that of free sialic acid. The inhibitory effect of free sialic acid and glycan sialic acid on tyrosinase activity in B16 cells was shown in Figure 9B. The EC_50_ of free sialic acid and glycan sialic acid was 0.40 mg/mL and 1.89 mg/mL. The tyrosinase inhibitory activity of free sialic acid was significantly higher than that of glycan sialic acid.

#### 3.3.4. Analysis of Activity Contribution Rate of Sialic Acid and Protein Components in Digested EBN

The EC_50_ concentration of antioxidant activity and tyrosinase inhibitory activity of free sialic acid, glycan sialic acid and digested EBN are shown in Table 1. It was revealed that the dry basis contents of free sialic acid and glycan sialic acid in the digested EBN were 3.51% and 2.39%, respectively. The contribution rates of free sialic acid, glycan sialic acid, and protein components in the digested EBN were calculated by the ratio conversion of EC_50_ concentration. The results of cell antioxidant activity showed that protein was the main antioxidant component in the digested EBN, with a contribution rate of 85.87%, followed by free sialic acid, with a contribution rate of 11.97%, and glycan sialic acid was the lowest, with a contribution rate of only 2.16%. The results showed that free sialic acid (63.43%) was the main bioactive ingredient in the digested products; the contribution rate of protein components was only 27.43%.

## 4. Conclusions

EBN, as a traditional whitening health food in China, is rich in protein and sialic acid. The EBN protein is difficult to dissolve; more than 50% of the glycoprotein was still undissolved after the simulated digestion. The polypeptides in the digested products of EBN were mainly oligopeptides, while 34% of the water-soluble proteins after digestion were still not hydrolyzed into polypeptides. After gastrointestinal digestion, 17.82% of the sialic acid of the stewed EBN was present in free form, 12.24% was bound to glycans, 15.39% was bound to proteins, and 54.55% was still insoluble in the digestion supernatant. Therefore, the overall digestion and utilization of sialic acid were low. The whitening activity of the hydrolysates of EBN was evaluated by the antioxidant activity and tyrosinase inhibition activity, respectively. It was found that the main antioxidant component in the digestive products was protein, and the main component inhibiting tyrosinase activity was FFSA. It was also found that the antioxidant activity and tyrosinase inhibitory activity of the FFSA was higher than that of GFSA. The protein and sialic acid after the hydrolysis of EBN have different biological functions. The products of the complete digestion of EBN are suitable for the development of a new generation of whitening health products.

## Figures and Tables

**Table 1:** Activity contribution rate of sialic acid and protein components in digested EBN.

Sample	Free Sialic Acid	Glycan Sialic Acid	Digested EBN
Antioxidant activity EC_50_(mg/mL)	0.54	2.04	1.84
Tyrosinase inhibitory activity EC_50_(mg/mL)	0.4	1.89	7.22
Component	Free Sialic acid	Glycan Sialic acid	Protein Component
Contribution rate of antioxidant activity (%)	11.97	2.16	85.87
Contribution rate of tyrosinase inhibitory activity (%)	63.43	9.14	27.43

## Data Availability

Data are contained within the article.
